# Spectrum of pathogenic variants and high prevalence of pathogenic *BBS7* variants in Russian patients with Bardet–Biedl syndrome

**DOI:** 10.3389/fgene.2024.1419025

**Published:** 2024-07-18

**Authors:** M. Orlova, P. Gundorova, V. Kadnikova, A. Polyakov

**Affiliations:** ^1^ DNA-diagnostics Laboratory, Research Centre for Medical Genetics, Moscow, Russia; ^2^ University Children’s Research, University Medical Center Hamburg-Eppendorf, Hamburg, Germany

**Keywords:** Bardet–Biedl syndrome, ciliopathies, BBSome, BBS genes, rare diseases

## Abstract

**Introduction:**

Bardet–Biedl syndrome is a rare condition characterized by obesity, retinitis pigmentosa, polydactyly, development delay, and structural kidney anomalies. This syndrome has an autosomal recessive type of inheritance. For the first time, molecular genetic testing has been provided for a large cohort of Russian patients with Bardet–Biedl syndrome.

**Materials and methods:**

Genetic testing was provided to 61 unrelated patients using an MPS panel that includes coding regions and intronic areas of all genes (*n* = 21) currently associated with Bardet–Biedl syndrome.

**Results:**

The diagnosis was confirmed for 41% of the patients (*n* = 25). Disease-causing variants were observed in *BBS1, BBS4, BBS7, TTC8, BBS9, BBS10, BBS12*, and *MKKS* genes*.* In most cases, pathogenic and likely pathogenic variants were localized in *BBS1, BBS10*, and *BBS7* genes; recurrent variants were also observed in these genes.

**Discussion:**

The frequency of pathogenic and likely pathogenic variants in the *BBS1* and *BBS10* genes among Russian patients matches the research data in other countries. The frequency of pathogenic variants in the *BBS7* gene is about 1.5%–2% of patients with Bardet–Biedl syndrome, while in the cohort of Russian patients, the fraction is 24%. In addition, the recurrent pathogenic variant c.1967_1968delinsC was detected in the *BBS7* gene. The higher frequency of this variant in the Russian population, as well as the lack of association of this pathogenic variant with Bardet–Biedl syndrome in other populations, suggests that the variant c.1967_1968delinsC in the *BBS7* gene is major and has a founder effect in the Russian population. Results provided in this article show the significant role of pathogenic variants in the *BBS7* gene for patients with Bardet–Biedl syndrome in the Russian population.

## Introduction

Bardet–Biedl syndrome is a rare autosomal recessive genetic ciliopathy. Its prevalence is estimated to be approximately 1:140000–160000 (Europe and North America) (Katsanis et al., 2001; Forsythe and Beales, 2013), but it can be higher in some isolated populations (Farag and Teebi, 1989). Bardet–Biedl syndrome is characterized by retinitis pigmentosa, obesity, polydactyly, hypogonadism, renal malformations, development delay, and intellectual disability.

Some research suggests that Bardet–Biedl syndrome may exhibit a complex pattern of inheritance, deviating from the classical Mendelian inheritance model (Perea-Romero et al., 2022). To date, triallelic ciliary gene mutations do not seem to make a significant contribution to the phenotype. Most of the available BBS patients are biallelic mutation carriers (Hichri et al., 2005a; Billingsley et al., 2011; Ece Solmaz et al., 2015), while other genetic types of the disease are described in very small patient series or even in single families.

Ciliopathies are a group of genetic disorders characterized by structural or functional defects in cilia. The group of ciliopathies also includes Joubert, Alstrom, Senior-Loken, Kartagener, Meckel, McKusick–Kaufman syndromes, and Leber congenital amaurosis.

Cilia are microtubule organelles localized on a cell surface. There are motile and non-motile types of cilia. Motile cilia functions are mucociliary clearance, fluid liquor in ventricles, and fallopian tubes. Non-motile cilia are present on almost all human cell types and act like olfactory and photoreceptors. It is well known that cilia are necessary for cell signaling; they play an important role in proper organism development and functioning (Mitchison and Valente, 2017).

The foundation of cilia is a basal body. It needs to form axonemal microtubules. The main cytoskeleton is composed of the axoneme. There is a transition zone between the basal body and the axoneme, which transforms basal body microtubes to the axoneme. The axoneme plays a role in intraflagellar transport (IFT). IFT provides two types of transport: anterograde, which transports signaling molecules on the top of cilia, and retrograde, which removes molecules from the top of cilia. On the top of the cilia, anterograde transport switches to retrograde transport. The cilia membrane is a continuation of the cytoplasmic membrane (Fliegauf et al., 2007).

One of the key components for proper cilia function is the BBSome, an octameric protein complex composed of BBS1, BBS2, BBS4, BBS5, BBS7, TTC8, BBS9, and BBIP1 proteins (Hichri et al., 2005b). The BBSome links transport proteins (dyneins and kinesins) with signaling molecules delivered to and removed from the cilia surface.

To date, 21 genes are known to be associated with Bardet–Biedl syndrome. In addition to the aforementioned proteins that form the BBSome itself, the proteins responsible for BBSome formation, regulatory proteins, and cilia formation are very important (Ece Solmaz et al., 2015).

The most frequent pathogenic changes are localized in genes *BBS1* (22%–23%), *BBS10* (20–28.6%), *BBS2* (8–15.9%), and *BBS12* (5–14.3%) (Deveault et al., 2011; Forsythe and Beales, 2013). A common nucleotide variant, с.1169T>C, which led to missense p. Met390Arg, has been identified in the *BBS1* gene (Mykytyn et al., 2002). In addition, recurrent pathogenic variants are described in the *BBS10* gene: c.271dup, which lead to frameshift and premature stop-codon formation p. Cys91LeufsTer5 (Lindstrand et al., 2014; Sathya Priya et al., 2015), and SNV c.145C>T, which leads to missense variant p. Arg49Trp (Lindstrand et al., 2014).

The aim of this study was to investigate the major disease-causing genes and pathogenic variant spectrum in Russian patients with a clinical diagnosis of Bardet–Biedl syndrome.

## Materials and methods

The biological material was collected in laboratory rooms of medical genetic counseling institutions from various regions of the Russian Federation. Informed consent was obtained from all patients (or their parents/guardians for underage patients). The whole venous blood samples were collected into single-use plastic test tubes containing an anticoagulant (EDTA). DNA was extracted from peripheral blood leukocytes using the Wizard^®^ Genomic DNA Purification Kit (Promega, Madison, WI, United States), according to the manufacturer’s protocol (Wizard Genomic DNA Purification Kit, 2023).

We performed MPS panel sequencing on 61 unrelated patients with Bardet–Biedl syndrome. The custom-made MPS panel includes coding regions and intronic areas of all 21 genes known to be associated with Bardet–Biedl syndrome today. For the first time, molecular genetic testing was provided for the cohort of 61 Russian patients from unrelated families with Bardet–Biedl syndrome.

In addition to the genes directly associated with Bardet–Biedl syndrome (*BBS1, BBS2, ARL6, BBS4, BBS5, MKKS, BBS7, TTC8, BBS9, BBS10, TRIM32, BBS12, MKS1, CEP290, WDPCP, SDCCAG8, LZTFL1, BBIP1, IFT27, IFT172,* and *C8orf37*), the panel also included genes associated with other ciliopathies, such as Alstrom, Joubert, Meckel, and Cohen syndromes and other ciliopathies (Table 1).

**TABLE 1:** List of phenotypes and associated genes included in the panel.

Phenotype	Number of genes	Genes
Bardet–Biedl syndrome	21	*BBS1, BBS2, ARL6, BBS4, BBS5, MKKS, BBS7, TTC8, BBS9, BBS10, TRIM32, BBS12, MKS1, CEP290, WDPCP, SDCCAG8, LZTFL1, BBIP1, IFT27, IFT172, C8orf37*
Joubert syndrome	32	*AHI1, ARL13B, ARMC9, B9D1, B9D2, C5orf41, CC2DA, CEP104, CEP120, CEP290, CEP41, CSPP1, INPP5E, KIAA0556, KIAA0586, KIF7, MKS1, NPHP1, OFD1, PDE6D, PIBF1, RPGRIP1L, TCTN1, TCTN2, TCTN3, TMEM107, TMEM138, TMEM216, TMEM231, TMEM237, TMEM67, ZNF423*
Meckel syndrome	11	*B9D1, B9D2, CC2D2A, CEP290, MKS1, RPGRIP1L, TCTN2, TMEM107, TMEM216, TMEM231, TMEM67*
Retinitis pigmentosa	5	*ARL6, C8orf37, IFT172, OFD1, TTC8*
Orofaciodigital syndrome	5	*C5orf42, KIAA0753, OFD1, TCTN3, TMEM107*
Short-rib thoracic dysplasia	4	*CEP120, IFT172, KIAA0586, TTC21B*
Nephronophthisis	4	*NPHP1, TMEM67, TTC21B, ZNF423*
COACH syndrome	3	*CC2D2A, RPGRIP1L, TMEM67*
Senior–Loken syndrome	3	*CEP290, NPHP1, SDCCAG8*
Cone-rod dystrophy	1	*C8orf37*
Leber congenital amaurosis	1	*CEP290*
Mental retardation, truncal obesity, retinal dystrophy, and micropenis	1	*INPP5E*
Hydrolethalus syndrome	1	*KIF7*
Acrocallosal syndrome	1	*KIF7*
McKusick–Kaufman syndrome	1	*MKKS*
Simpson–Golabi–Behmel syndrome	1	*OFD1*
RHYNS syndrome	1	*TMEM67*
Muscular dystrophy, limb-girdle, autosomal recessive	1	*TRIM32*
Cohen syndrome	1	*VPS13B*
Congenital heart defects, hamartomas of tongue, and polysyndactyly	1	*WDPCP*

Preparation of DNA libraries was performed using the AmpliSeq™ Library Kit (Thermo Fisher Scientific, United States), based on ultramultiplex PCR technology, according to the manufacturer’s protocol. Preparation of a sample for sequencing, which includes emulsion PCR and loading of the resulting microspheres onto the chip, was performed automatically using the Ion Shef system (Thermo Fisher Scientific, United States). Sequencing was performed on the Ion S5 system.

Mean panel coverage depth–628×, mean uniformity–97.78%.

Data analysis, variant calling, and interpretation were performed using the “NGSData” application developed by the Research Centre for Medical Genetics bioinformatics department (Beskorovayniy and Beskorovaynaya, 2024).

All identified variants were described using the HGVS nomenclature. The Genome Aggregation Database (gnomAD) was used to assess the population frequencies of the variants. The clinical significance of the variants was analyzed using the following resources: Online Mendelian Inheritance in Man^®^ (OMIM), Disease-Causing Mutations database HGMD^®^ Professional, and literature data. Assessment of the pathogenicity of the identified variants was based on Russian Guidelines for the interpretation of massive parallel sequencing variants (Ryzhkova et al., 2019).

To analyze the expected differences between allelic frequencies of pathogenic variants in Russian population and populations of other countries, the nonparametric сhi-squared test was used. The differences were considered statistically significant when *p* < 0.05.

## Results

The 61 patients with Bardet–Biedl syndrome enrolled in this study were screened using a custom panel of 21 genes associated with the disease. Total variants were observed on 86 chromosomes (Table 2). Pathogenic variants were detected on 35 chromosomes, likely pathogenic variants were detected on 23 chromosomes, and variants of unknown significance were detected on 28 chromosomes. We did not find variants in genes associated with other ciliopathies; we only found variants in those genes associated with Bardet–Biedl syndrome.

**TABLE 2:** List of variants detected in a cohort of Bardet–Biedl syndrome patients. Het–heterozygous, hom–homozygous, VUS–variant of unknown significance. Variants leading to diagnosis confirmation are written in bold.

Gene	Patient ID	cDNA	Effect	Zygosity	Clinical significance	References
*BBS1* *NM_024649.4*	**13**	**c.830+1G>T**	**splicing**	het	Likely pathogenic	Fauser et al. (2003), Xiong et al. (2015)
		**c.1318C>T**	**p.Arg440Ter**	het	Likely pathogenic	Hichri et al. (2005b), Mykytyn et al. (2003)
	17	c.1110 + 30_1110 + 33del		het	VUS	This study
	18	c.159 + 23G>A		het	VUS	This study
	**35**	**c.1717C>T**	**p.Gln573Ter**	hom	Likely pathogenic	This study
	**79**	**c.479+2T>G**	**splicing**	het	Pathogenic	This study
		**c.1401C>G**	**p.Tyr467Ter**	het	Likely pathogenic	This study
	**85**	**c.479+2T>G**	**splicing**	hom	Pathogenic	This study
	**41**	**c.479G>A**	**p.Arg160Gln**	het	Pathogenic	Hichri et al. (2005b)
		**c.667del**	**p.Thr223ProfsTer17**	het	Likely pathogenic	This study
	44	с.1169T>C	p.Met390Arg	het	Pathogenic	Mykytyn et al. (2002)
	68	с.1169T>C	p.Met390Arg	het	Pathogenic	Mykytyn et al. (2002)
*BBS2* *NM_031885.3*	3	c.943C>T	p.Arg315Trp	het	Pathogenic	Katsanis et al. (2001), Zaghloul et al. (2010)
		c.926C>T	p.Ser309Leu	het	VUS	This study
	14	c.653G>A	p.Gly218Asp	het	VUS	this study
		c.72C>G	p.Tyr24Ter	het	Pathogenic	Katsanis et al. (2001)
	81	c.194A>T	p.Asp65Val	het	VUS	This study
*BBS4* *NM_033028.4*	**67**	**c.1106+2T>A**	**splicing**	hom	Pathogenic	Yates et al. (2017), Bin et al. (2009)
*BBS5* *NM_152384.2*	**9**	**c.619-1G>C**	**splicing**	hom	Likely pathogenic	Xiong et al. (2015), Feuillan et al. (2011)
	87	c.208 + 5G>A	splicing	hom	VUS	This study
*CEP290* *NM_025114.3*		c.6596C>T	p.Thr2199Ile	het	VUS	This study
		c.3154A>G	p.Ile1052Val	het	VUS	This study
	23	c.7015C>T	p.Arg2339Trp	het	VUS	This study
*BBS7* *NM_176824.2*	**4**	**c.1773del**	**p.Asn592ThrfsTer6**	hom	Likely pathogenic	This study
	5	c.718 + 3A>G		hom	VUS	This study
	6	c.1583T>G	p.Leu528Arg	het	VUS	This study
		c.712_715del	p.(Arg238GlufsTer59)	het	Pathogenic	Bin et al. (2009)
	**10**	**c.1967_1968delinsC**	**p.(Leu656ProfsTer18)**	hom	Pathogenic	Muller et al. (2010)
	15	c.1967_1968delinsC	p.(Leu656ProfsTer18)	het	Pathogenic	Muller et al. (2010)
		c.790G>A	p.Gly264Arg	het	VUS	Weisschuh et al. (2020)
	**21**	**c.1967_1968delinsC**	**p.(Leu656ProfsTer18)**	hom	Pathogenic	Muller et al. (2010)
	24	c.1967_1968delinsC	p.(Leu656ProfsTer18)	het	Pathogenic	Muller et al. (2010)
		c.455G>T	p.Cys152Phe	het	VUS	This study
	**46**	**c.1062_1063del**	**p.Tyr354Ter**	het	Likely pathogenic	Riedhammer et al. (2020)
		**c.712_715del**	**p.Arg238GlufsTer59**	het	Pathogenic	Bin et al. (2009)
	**42**	**c.1967_1968delinsC**	**p.(Leu656ProfsTer18)**	hom	Pathogenic	Muller et al. (2010)
	**58**	**c.1967_1968delinsC**	**p.(Leu656ProfsTer18)**	het	Pathogenic	Muller et al. (2010)
		c.712_715del	p.Arg238GlufsTer59	het	Pathogenic	Bin et al. (2009)
	92	c.495G>C	p.Leu165Phe	hom	VUS	This study
	**31**	c.1230 + 5G>A		het	VUS	This study
*BBS10* *NM_024685.3*		**c.959_962del**	**p.(Ser320IlefsTer5)**	hom	Likely pathogenic	Shaheen et al. (2016)
	**2**	**c.1024dup**	**p.Ile342AsnfsTer20**	het	Pathogenic	Ece Solmaz et al. (2015)
		**c.271dup**	**p.Cys91LeufsTer5**	het	Pathogenic	Lindstrand et al. (2014)
	**16**	**c.145C>T**	**p.Arg49Trp**	het	Pathogenic	Lindstrand et al. (2014)
		**c.271dup**	**p.Cys91LeufsTer5**	het	Pathogenic	Lindstrand et al. (2014)
	22	c.271dup	p.(Cys91LeufsTer5)	het	Pathogenic	Lindstrand et al. (2014)
		c.-151T>C		het	VUS	This study
	**30**	**c.539G>A**	**p.Gly180Glu**	het	Pathogenic	Janssen et al. (2011)
		**c.271dup**	**p.Cys91LeufsTer5**	het	Pathogenic	Lindstrand et al. (2014)
	**40**	**c.145C>T**	**p.Arg49Trp**	hom	Pathogenic	Lindstrand et al. (2014)
	68	c.1969A>G	p.Thr657Ala	het	VUS	This study
	**80**	**c.271dup**	**p.Cys91LeufsTer5**	hom	Pathogenic	Lindstrand et al. (2014)
*TTC8* *NM_198309.3*	59	c.459G>A	p.Thr153Thr	het	Pathogenic	Stoetzel et al. (2006)
		c.651G>T	p.Trp217Cys	het	VUS	This study
	28	c.128T>C	p.Leu43Ser	hom	VUS	This study
	**82**	**c.871_875del**	**p.Ile301Ter**	hom	Likely pathogenic	This study
*BBS9* *NM_198428.2*	**74**	**c.677_678dup**	**p.Leu227AsnfsTer8**	hom	Likely pathogenic	This study
*BBS12* *NM_152618.2*	11	c.68C>T	p.Ala23Val	het	VUS	This study
	**57**	**c.376G>T**	**p.Glu126Ter**	het	Likely pathogenic	This study
		**c.2023C>T**	**p.Arg675Ter**	het	Pathogenic	Dulfer et al. (2010)
	91	c.1792T>G	p.Tyr598Asp	hom	VUS	This study
*MKKS* *NM_018848.3*	**51**	**c.1273-5_1273-2delinsAAAG**	**splicing**	het	Likely pathogenic	This study
		**c.110A>G**	**p.Tyr37Cys**	het	Pathogenic	Muller et al. (2010), Scheidecker et al. (2015)
	**60**	**c.732_733del**	**p.Phe244LeufsTer10**	hom	Likely pathogenic	This study
	65	c.802C>T	p.Leu268Phe	het	VUS	This study
*IFT4* *NM_025103.2*	8	c.1624-1G>A	splicing	het	Likely pathogenic	This study
*WDPCP* *NM_015910.5*	20	c.220G>C	p.Gly74Arg	het	VUS	This study

Two pathogenic or likely pathogenic variants were detected in 25 of 61 patients, confirming the molecular genetic diagnosis of Bardet–Biedl syndrome in 41% of the patients. Our results support a biallelic pattern of inheritance of Bardet–Biedl syndrome.

Variants of unknown significance were detected in 23 patients. We could not find a genetic cause of disease for 13 patients.

**FIGURE 1:**
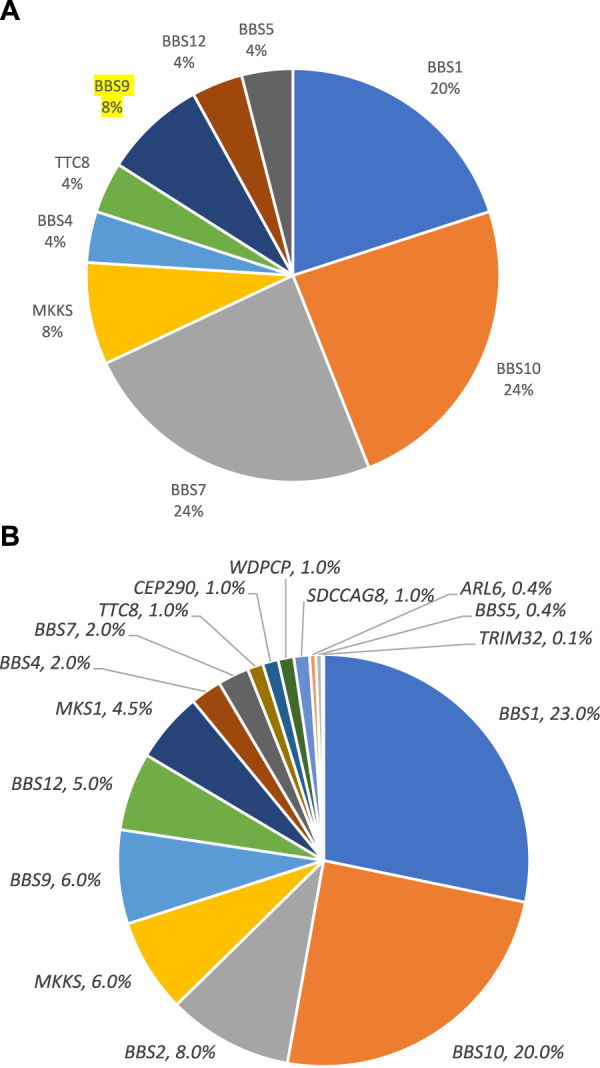
Distribution of variants in genes in patients with confirmed diagnosis. **(A)**. This study. **(B)**. Literature data (Forsythe and Beales, 2013).

**FIGURE 2:**
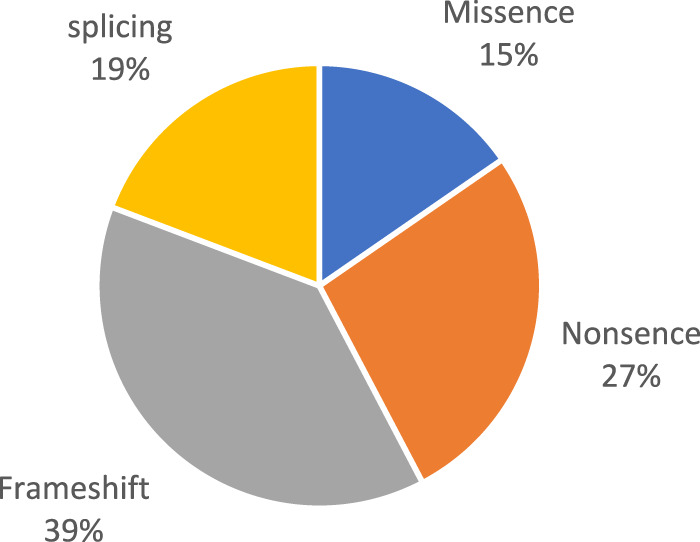
Types of detected variants.

Pathogenic and likely pathogenic variants were observed in the following genes: *BBS1, BBS4, BBS7, TTC8, BBS9, BBS10, BBS12,* and *MKKS* (Figure 1). All of these genes are associated with Bardet–Biedl syndrome. The prevalence variants are frameshift (Figure 2). In most cases, pathogenic and likely pathogenic variants were found in the *BBS1, BBS10,* and *BBS7* genes. Recurrent variants were detected in these three genes (Table 3).

**TABLE 3:** Recurrent pathogenic variants identified in patients with a confirmed diagnosis.

Gene	Variant	Number of chr. with this variant	Number of chr. with P and LP variants in this gene	Share of chr. with P and LP variants in this gene	GnomAD allele freq.^1^	RuExac allele freq.^2^	*p*-value*
*BBS1*	c.479 + 2T>G	3	10	0.3	0.0000956	0	0
*BBS7*	c.1967_1968delinsC	7	12	0.583333333	0.00008843	0.0015	**p < 0.001**
*BBS10*	c.271dup	5	13	0.3846153846	0.0005642	0.0011	*p* > 0.5
	c.145C>T	3	13	0.2307692308	0.00005371	0	0

^a^ Genome Aggregation Database (gnomAD v.2.1.1) allele frequencies provided on 125,748 exomes and 15,708 genomes.

^b^ Database of Russian population allele frequencies (RuExac) provided on 1,337 exomes Nucleotide variants database, 2024.

^c^ Differences between allelic frequencies of pathogenic variants in the Russian population and populations of other countries were analyzed by the сhi-squared test.

The frequency of pathogenic and likely pathogenic variants in the *BBS1* and *BBS10* genes among Russian patients was similar to the frequencies reported in other populations (Deveault et al., 2011; Forsythe and Beales, 2013). However, the rate of pathogenic and likely pathogenic variants in the *BBS7* gene was much higher in the Russian patients, reaching 24% (Figure 1A), compared to the reported frequency of approximately 1.5%–3% in other populations (Katsanis, 2004; Muller et al., 2010; Deveault et al., 2011). The most notable finding was the recurrent pathogenic variant c.1967_1968delinsC (p.(Leu656ProfsTer18)) in the *BBS7* gene, detected in four Russian patients, two in a homozygous and two in a heterozygous state. This variant accounted for 58% of all pathogenic and likely pathogenic variants identified in the *BBS7* gene in the Russian cohort. The allele frequency of this variant in the Russian population (0.15%) was significantly higher (*p* < 0.001) than in other populations, such as the European population (0.019%) and the African population (0.004%), according to the gnomAD database. This suggests that the c.1967_1968delinsC variant in the *BBS7* gene is a major founder pathogenic variant in the Russian population.

In the *BBS1* gene, a novel variant localized in the canonical splice site c.479 + 2T>G was found. This variant was observed in two patients: patient 85 in homozygous state and patient 79 in heterozygous state with novel nonsense-variant c.1401C>G (p.Tyr467Ter). *In silico* algorithms SpliceAI, Human Splicing Finder, FSPLICE, and MutationTaster classified variant c.479 + 2T>G as possibly affecting splicing.

In five of seven patients with pathogenic variants of the *BBS10* gene, common variants c.271dup (p.Cys91LeufsTer5) (Lindstrand et al., 2014; Sathya Priya et al., 2015) and c.145C>T (p.Arg49Trp) (Deveault et al., 2011; Scheidecker et al., 2015) were detected.

## Discussion

The high rate of novel, previously unreported variants identified in this study emphasizes the need for further investigation of the molecular genetic basis of Bardet–Biedl syndrome, especially in understudied populations.

Currently, the MPS gene panel has proven to be effective in diagnosing Bardet–Biedl syndrome. The detection rate is 41%. The panel includes the coding sequences of all currently known genes (*n* = 21) associated with Bardet–Biedl syndrome, which makes it an effective and comprehensive tool for molecular genetic diagnosis of this pathology.

Our research shows that in 25 patients (41%), pathogenic or likely pathogenic variants are present in homozygous or compound heterozygous states, which allows confirming the diagnosis by the molecular genetic method. This rate is slightly lower than the 60%–87% reported in the literature (Muller et al., 2010; Deveault et al., 2011; Ece Solmaz et al., 2015; Manara et al., 2019) and may be due to the broader inclusion criteria used in this study, with patients not necessarily presenting the full Bardet–Biedl syndrome phenotype. The mean panel coverage depth (628×) and uniformity (97.78%) suggest that technical issues are unlikely to be the cause. However, the Ion S5 system is known to have limitations in covering homopolymer regions, potentially leading to missed variants. We believe that the lower detection rate is primarily due to the characteristics of our patient cohort.

Variants of unknown significance were detected in 23 patients.

If family analyses were provided, these variants could be related to phenotype; they might also be characterized through functional analysis or the appearance of literature data showing a significant pathogenic role of the variant.

Further description of variants is based on data obtained from DNA samples from patients diagnosed with Bardet–Biedl syndrome in this study using the MPS panel of genes.

The present research shows that the most common pathogenic variants are localized in genes *BBS1, BBS7*, and *BBS10*. The frequency of pathogenic and likely pathogenic variants in the genes *BBS1* and *BBS10* among Russian patients matches the frequencies in other populations.

According to literature data, the frequency of pathogenic variants in *BBS7* is approximately 1.5%–3% of the patients with Bardet–Biedl syndrome (Katsanis, 2004; Muller et al., 2010; Deveault et al., 2011). In the Turkish population, different pathogenic variants in this gene were found in 15% (2 of 13 patients), which can be explained by the isolation of the population (Muller et al., 2010). Meanwhile, the proportion is 24% in the cohort of Russian patients. This suggests that pathogenic variants in the *BBS7* gene, as well as pathogenic variants in genes *BBS1* and *BBS10,* are prevalent in the Russian population.

The most frequent variant in the *BBS1* gene is SNV, localized in canonical splice site c.479 + 2T>G. This variant is annotated as likely pathogenic in the Clinvar database (RCV000666424), but evidence details were not provided. In a cohort of patients with a confirmed diagnosis, chromosomes with this variant form 30% of all chromosomes with pathogenic and likely pathogenic variants in this gene. Meanwhile, the most common pathogenic variant worldwide, 1169T>C (p.Met390Arg), was not observed in a cohort of Russian patients with a confirmed diagnosis.

The frequency of pathogenic and likely pathogenic variants in the *BBS10* gene among Russian patients matches the research data in other countries. Panel sequencing in other countries shows that pathogenic variants in gene *BBS10* make up 20%–31% of cases; our research shows similar results at 24%.

Pathogenic variant c.271dup (p.Cys91LeufsTer5) was found on five of 13 chromosomes (38%) with pathogenic variants in gene *BBS10*. Pathogenic variant c.145C>T (p.Arg49Trp) was found on three of 13 chromosomes (23%). In different studies, these two variants were observed in 48%–54% (Muller et al., 2010; Billingsley et al., 2011) and 8%–10% (Muller et al., 2010; Billingsley et al., 2011) of their gene’s pathogenic alleles, respectively.

The important finding is the pathogenic variant c.1967_1968delinsC in gene *BBS7*, detected in four patients. This variant was reported as disease-causing in two related Russian patients with Bardet–Biedl syndrome (Suspitsin et al., 2015). The proportion of variant c.1967_1968delinsC in the *BBS7* gene from all chromosomes with pathogenic and likely pathogenic variants in this gene is 58%. The proportion of chromosomes carrying variant c.1967_1968delinsC relative to all chromosomes with pathogenic and likely pathogenic variants (n = 58) is 12%, which makes this variant the most common in the cohort of Russian patients, as well as pathogenic variant c.271dup in the *BBS10* gene.

Variant c.1967_1968delinsC in the *BBS7* gene was described in a heterozygous state with another variant in one European patient with Bardet–Biedl syndrome (no data about ethnicity were contributed), but no information supporting the pathogenicity of this variant was provided (Muller et al., 2010). In addition, this variant was described in a homozygous state in two Russian siblings. Their parents and third healthy child are heterozygous carriers. Researchers report finding this variant in a heterozygous state in 0.007% of 2832 healthy people (Suspitsin et al., 2015). In a database of population allele frequencies, the Genome Aggregation Database (gnomAD), variant c.1967_1968delinsC was detected in 0.0088% of people, while in database of Russian population allele frequencies, RuExac (based on 1337 exomes), this variant was detected in 0.15% of the people (Table 3). These people came from different regions of Russia. According to gnomAD, the allele frequency in the European population is 0.019%, and it is 0.004% in the African population. Variant c.1967_1968delinsC was not described in other populations.

We used a сhi-squared test to analyze differences between c.1967_1968delinsC allelic frequencies in a Russian population and populations of other countries. The chi-squared test confirmed a statistically significant (*p* < 0.001) increase in the frequency of this variant in the Russian population.

All four patients with pathogenic variant c.1967_1968delinsC in the *BBS7* gene of this study are of Russian descent from various regions of Russia. The higher frequency of this variant in the Russian population, as well as the lack of association of this pathogenic variant with Bardet–Biedl syndrome in other populations, suggests that variant c.1967_1968delinsC in the *BBS7* gene is major and has a founder effect in the Russian population.

## Data Availability

The datasets presented in this study can be found in online repositories. The names of the repository/repositories and accession number(s) can be found in the article.
